# MYC as a Multifaceted Regulator of Tumor Microenvironment Leading to Metastasis

**DOI:** 10.3390/ijms21207710

**Published:** 2020-10-18

**Authors:** Erna Marija Meškytė, Sabiha Keskas, Yari Ciribilli

**Affiliations:** 1 Laboratory of Molecular Cancer Genetics, Department of Cellular, Computational, and Integrative Biology (CIBIO), University of Trento, 38123 Povo (TN), Italy; ernamarija.meskyte@unitn.it (E.M.M.); louhad@outlook.com (S.K.); 2 Department of Biological Models, Institute of Biochemistry, Life Sciences Centre, Vilnius University, 10257 Vilnius, Lithuania; 3 Laboratory of Biology of Development and Differentiation, Department of Biology, University Oran 1 Es-Senia, Oran 31000, Algeria; 4 Department of Cellular Biology and Physiology, Faculty of Natural Life Sciences, University Blida 1, Ouled Yaïch 09015, Algeria

**Keywords:** MYC, TAMs, tumor microenvironment, metastasis, EMT

## Abstract

The *Myc* family of oncogenes is deregulated in many types of cancer, and their over-expression is often correlated with poor prognosis. The *Myc* family members are transcription factors that can coordinate the expression of thousands of genes. Among them, *c-Myc* (MYC) is the gene most strongly associated with cancer, and it is the focus of this review. It regulates the expression of genes involved in cell proliferation, growth, differentiation, self-renewal, survival, metabolism, protein synthesis, and apoptosis. More recently, novel studies have shown that MYC plays a role not only in tumor initiation and growth but also has a broader spectrum of functions in tumor progression. MYC contributes to angiogenesis, immune evasion, invasion, and migration, which all lead to distant metastasis. Moreover, MYC is able to promote tumor growth and aggressiveness by recruiting stromal and tumor-infiltrating cells. In this review, we will dissect all of these novel functions and their involvement in the crosstalk between tumor and host, which have demonstrated that MYC is undoubtedly the master regulator of the tumor microenvironment. In sum, a better understanding of MYC’s role in the tumor microenvironment and metastasis development is crucial in proposing novel and effective cancer treatment strategies.

## 1. Introduction

Myc is a family of proto-oncogenes that codes for transcription factors. The Myc family consists of three related human genes: *c-Myc (MYC), l-Myc (MYCL)*, and *n-Myc (MYCN)* [1,2]. MYC is one of the most influential transcription factors since it regulates at least 15% of the whole human genome. It is mainly involved in the regulation of cell cycle, proliferation, apoptosis, ribosome biogenesis, and metabolism. Under normal conditions, the expression of MYC is strictly controlled; however, in cancer, the activity of MYC is often deregulated, contributing to the initiation of tumorigenesis and maintenance of the disease [3,4,5]. Therefore, it is considered one of the most potent cellular oncogenes, and MYC over-expression is a frequent event in many types of human cancers. MYC activation can be direct through chromosomal translocation, genomic amplification, retroviral integration, and mutation. It can also be indirect since MYC can be activated through increased gene expression and protein stability by the activation of other oncogenes, including *RAS, SRC, NOTCH* or inactivation of tumor suppressor genes such as *APC* [2,6,7,8]. The over-expression of MYC has been almost invariably linked to tumorigenesis. Studies using inducible transgenic mouse models have demonstrated that MYC-induced tumors grow depending on the continuous expression of MYC [6,9]. The MYC family members are transcription factors that can coordinate the transcriptional expression of thousands of genes. MYC regulates the expression of its target genes through direct activation or inhibition of gene transcription, transcriptional amplification, the induction of microRNA and chromatin regulators, as well as the global regulation of RNA and protein biogenesis [2,8]. Canonically, MYC regulates the expression of genes involved in cell proliferation, growth, differentiation, self-renewal, survival, metabolism, protein synthesis, and apoptosis [10]. More recently, novel studies have shown that MYC can be considered a crucial regulator of tumor microenvironment [11,12]. MYC also promotes tumor progression, and it is often involved in the processes of resistance to chemotherapy and metastasis [13,14].

**Figure 1:**
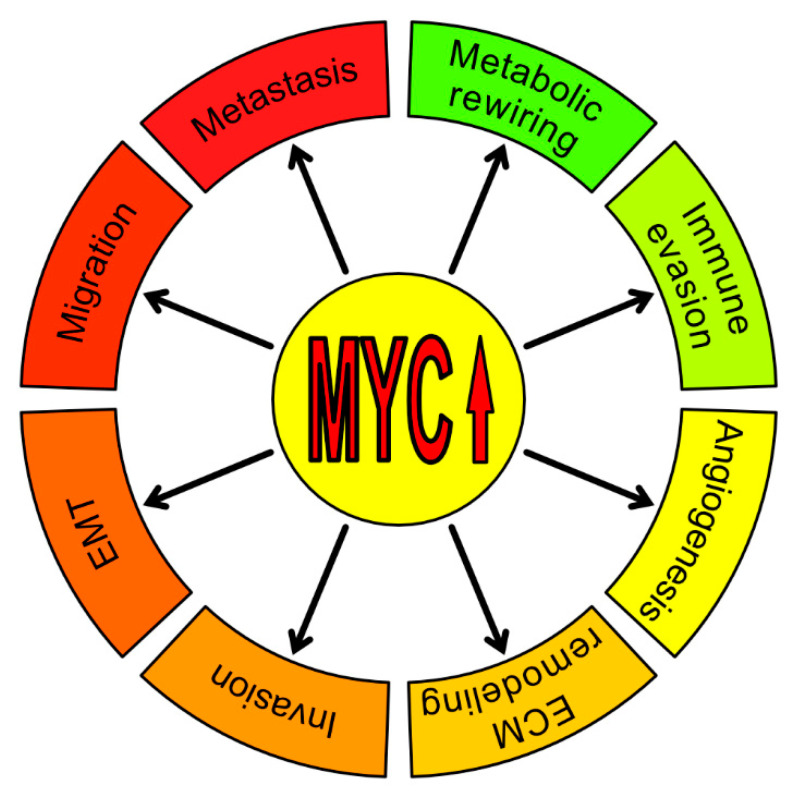
The role of MYC in cancer aggressiveness and progression. An increasing number of studies show that, besides the canonical functions such as cell proliferation, growth, differentiation, self-renewal, survival, metabolism, protein synthesis, and apoptosis, MYC is also directly or indirectly involved in other processes necessary for tumor progression—metabolic rewiring, immune evasion, angiogenesis, ExtraCellular Matrix (ECM) remodeling and invasion, migration, Epithelial-to-Mesenchymal Transition (EMT) and metastasis.

This review will focus on the direct and indirect impact of MYC in cancer aggressiveness and progression. It will also discuss the roles of MYC in regulating tumor microenvironment and metastasis formation. In Figure 1 we summarize the non-classical ways by which MYC is directly or indirectly involved in tumor progression.

## 2. MYC–A Key Player in the Tumor Microenvironment (TME)

The development and progression of tumors is a complex process that is not only affected by genetic events altering the biology of the cells which undergo transformation, but it is also greatly influenced by the surrounding microenvironment. Different studies showed that the tumor microenvironment (TME) is important for cancer initiation and the promotion of neoplastic growth. The tumor microenvironment is comprised of proliferating tumor cells and the tumor stroma, consisting of blood vessels, pericytes, a variety of associated tissue cells as well as infiltrating immune cells, belonging both to the innate and adaptive immune system [15,16,17]. It is now clear that oncogenes are not only responsible for uncontrolled cell division and intracellular signaling, but they also play a crucial role in instructing the tumor microenvironment [18,19]. Although it is well known that MYC is able to induce genomic instability, a fundamentally important feature of cancer cells, tumor microenvironment can also induce important genetic alterations in the surrounding cells, demonstrating that MYC and TME are allies in cancer progression [12,20,21,22]. Additional evidence demonstrates that MYC is also involved in the recruitment of different elements of tumor microenvironment: from making tumor stroma more accommodating for tumor cells to helping evade immune responses to push a tumor to a more invasive and metastatic phenotype [8,12,23,24,25].

### 2.1. Tumor-Infiltrating Cells within Tumor Microenvironment: Why MYC Matters?

Tumor-infiltrating cells include macrophages, endothelial cells, fibroblasts, T and B cells, neutrophils, Natural Killer (NK) cells, and others. All of these types of cells are involved in various tumor-promoting processes, such as immune response evasion, angiogenesis, metabolic changes, invasion and metastasis [26,27,28,29]. Moreover, pilling studies show that MYC is heavily involved in educating tumor-infiltrating cells and making them participate in tumor progression [24,30,31]. In this part of the review, we will concentrate on the cells within microenvironment recruited by MYC and how they crosstalk with cancer cells to evade the immune response, promote angiogenesis as well as bolster tumor invasion.

### 2.2. TAMs–Main Players in MYC-Regulated Tumor Microenvironment

Macrophages are immune cells that are typically involved in many different processes, such as the killing of pathogens, inflammation and its resolution, tissue remodeling, and angiogenesis. Unfortunately, macrophages are also involved in tumor growth [32]. Tumor-associated macrophages (TAMs) make a significant part (up to 50%) of the infiltrate of most, if not all, tumors [33,34,35]. Macrophage population is highly heterogeneous; they originate from circulating monocytic precursors and differentiate into distinct macrophage types. These subclasses are identified as M1 (or classically-activated) and M2 (or alternatively-activated) macrophages [34,36,37,38,39]. The tumor microenvironment pushes macrophages towards a wound healing/regulatory state that resembles several aspects of an alternatively-activated macrophage phenotype (M2-like) [40]. Tumor-associated macrophages share many similarities with prototypic polarized M2 macrophages, in terms of gene expression and functions. Knowing the various functions of M2 macrophage population, it is now generally accepted that TAMs promote tumor progression and metastasis by activating circuits that regulate tumor growth, adaptive immunity, anti-tumor immunity, stroma formation, and angiogenesis [32,35,41,42,43,44,45]. In a variety of human tumors, including breast, prostate, renal, bladder, lung, cervical carcinoma, glioma, lymphoma, and melanoma, a high number of TAMs infiltrating the tumor stroma is also associated with a higher incidence of metastasis and more aggressive types of cancer that eventually lead to a poor prognosis of the disease [46,47,48,49,50,51,52,53]. The transcription factor MYC is known to be involved in cell proliferation, apoptosis, tissue remodeling, angiogenesis, cell metabolism, and the production of both inflammatory and anti-inflammatory cytokines [54,55,56,57]. We can clearly see that the processes where MYC is linked considerably overlap the ones related to macrophages. In fact, a study by Pello and colleagues demonstrates that MYC is expressed by human-derived macrophages classed as M2 macrophages associated with anti-inflammatory functions, angiogenesis, and tissue remodeling. Additionally, it controls the expression of around 50% of alternative-specific markers, showing the role of MYC in macrophage polarization [24]. Indeed, it is important to mention that in human macrophages, MYC expression is restricted to the M2 phenotype, while MYC is not detected in M0 (resting) or M1 (pro-inflammatory) macrophages [58]. Jablonski and collaborators also identified MYC as a marker for murine M2 macrophages population [59]. MYC is expressed in TAMs as well, where it controls the expression of pro-tumor genes [24,30,60]. Pello and colleagues reported that MYC expression is induced in human macrophages by exposure to IL-4 and M2-like stimuli and is involved in the stimulation of a large set of genes during alternative macrophage activation – directly or indirectly via induction of signal transducer and activator of transcription-6 (STAT6) and peroxisome proliferator-activated receptor-γ (PPARγ) [24]. Furthermore, it has been reported that MYC is involved in the increased expression of a distinct subset of alternative macrophage activation genes, such as SCAvenger receptor class B type 1 (*SCARB1*), lipid-peroxiding enzyme 12/15—lipoxygenase (*ALOX15*), mannose receptor C type 1 (*MRC1*), cathepsin C (*CTCSC*), *CD209*, *PPARγ*, the A kinase anchor protein 12 (*AKAP12*), the monoamine oxidase A (*MAOA*) and the wingless-type MTV integration site family member 5A (*WNT5A*) [24,30,61]. MYC is also involved in the slight induction of *STAT6* [24]. The transcription of some of these target genes is more significantly induced upon MYC activation, like *ALOX15* or *CD209*, while for others even the basal levels of MYC may be sufficient for their expression [24]. Knowing that M2 macrophage population has similarities with tumor-associated macrophages, it was also important to investigate the role of MYC in the biology of TAMs. In experimental conditions, TAMs can be mimicked using simplified models, where macrophages are exposed to a conditioned medium derived from a cancer cell line [62,63]. Using this system, it has been demonstrated that exposure of macrophages to cancer cells conditioned medium induced the expression of MYC, as well as alternative activation markers including ALOX15, CD209, and MRC1. What is more, it is shown that MYC also promote the expression of *MMP9*, *VEGF*, *HIF-1α*, and *TGF-β* (all genes associated with cancer aggressiveness) in TAM. These findings were confirmed using mouse models as well [24,30]. It is additionally reported that TAM infiltration in neuroblastomas is associated with the induction of MYC expression via IL-6/STAT3 pathway, showing that there are putative feedback loop mechanisms in regulating the expression of MYC in the tumor microenvironment [60].

Several of these MYC-induced factors are reported to play an important role in TAM-dependent tumor progression and are summarized in Table 1. By producing growth factors, proteolytic enzymes and various inhibitory immune checkpoint proteins in T cells, TAMs have a major function in regulating tumor progression and metastasis [29]. We know that MYC stimulates specific expression programs in tumor-associated macrophages, but how exactly does it help cancer to progress? To answer this compelling question, we propose that MYC-induced proteins mainly participate in three processes: angiogenesis, immunosuppression and invasion/migration.

### 2.3. Angiogenesis–MYC Controls the “Angiogenic Switch”

In this section, we will describe the role of MYC in angiogenesis, with a particular focus on TAMs–as they are thought to be leading players in MYC-driven angiogenesis, as well as other reported mechanisms influenced by MYC up-regulation.

Tumor vascularization or angiogenesis is one of the hallmarks of cancer and plays a crucial role in tumor growth, invasion, and formation of metastasis [74,75]. During tumor progression, the network of blood vessels keeps increasing — this process also known as the “angiogenic switch” [76,77,78]. Many molecules and different types of cells are involved in tumor angiogenesis, and various pro-angiogenic factors are secreted by both cancer and tumor-infiltrating cells [76,77,78]. In the past 20 years, several reports suggest that TAMs are critical players in regulating the “angiogenic switch” and are essential in stimulating tumor angiogenesis and progression [29,79,80]. It has also been noted that the up-regulation of MYC was found in cancer-associated fibroblasts (CAFs) and that CAFs can promote tumor angiogenesis by secreting cytokines that recruit endothelial cells to form tumor-associated blood vessels—suggesting that MYC is also important in the regulation of CAFs within tumor microenvironment [81,82,83]. Hypoxia is recognized as a major driver of angiogenesis. Tumor-infiltrating macrophages accumulate in hypoxic areas of the tumor [79]. Kortlever and colleagues report that MYC activation triggers the abrupt onset of angiogenesis, marked by loss of vessel integrity, increased vessel leakiness, and relief of the widespread hypoxia, characteristic of the original indolent adenomas [18].

Hypoxia-inducible factor 1-alpha (HIF-1α) and vascular endothelial growth factor (VEGF) are two critical elements in tumor angiogenesis, and both are up-regulated upon MYC activation both in tumor cells and TAMs [17,24,65]. HIF-1α modulates the recruitment of macrophages to hypoxic regions of the tumor and regulates the expression of many genes involved in tumor angiogenesis [79,84,85]. Relatively recent studies show that MYC activation lead to the up-regulation of VEGF and, consequently, blood vessel permeability and loss of integrity; hence, VEGF is thought to be the principal instigator of MYC-induced lung adenoma angiogenesis. Following MYC activation, VEGF was highly induced in TAMs, suggesting that macrophages are the primary angiogenic mediators [18,86,87]. Via VEGF-C/VEGFR-3 signaling, TAMs also account for lymphangiogenesis - an essential route for cancer cells dissemination to regional lymph nodes and distant metastasis [29,86].

VEGF released by tumor and surrounding cells initiates the angiogenic process by activating endothelial cells and promoting their sprouting and migration. VEGF mainly binds to two related receptors on endothelial cells, VEGF receptor 1 (also known as Flt-1) and VEGF receptor 2 (also known as Flk-1). Binding and activation of VEGF receptors lead to endothelial cell survival, proliferation, and migration, hence supporting and sustaining angiogenesis [88,89]. In addition to its pro-angiogenic effects, VEGF also stimulates the growth of tumors by inducing TAMs infiltration and by stimulating M2 polarization, when IL-4 and IL-10 are present [90,91].

It has been reported that MYC also regulates the expression of other genes involved in angiogenesis. Studies in non-small cell lung cancer, show that MYC up-regulates kininogen, a hemostatic factor which functions as an angiogenic factor through the signaling of its cleaved product from bradykinin via the B receptor [92,93]. The same researchers showed that MYC induced the G-protein-coupled receptor Adora2b. Adenosine A2B receptor (Adora2b) stimulates angiogenesis by inducing VEGF and the endothelial nitric oxide synthase (eNOS) [93,94]. Furthermore, in all subtypes of lung tumors, the expression of Sema3F was found significantly reduced [93,95]. Semaphorin-3F (Sema3F) is known as a negative regulator of tumor angiogenesis. Semaphorin-3F inhibits HIF-1α and, consequently, VEGF expression, which causes the block of hypoxia-induced angiogenesis [96,97]. Other studies show that MYC activates angiogenesis by increasing the expression of Interleukin 1β and VEGF and suppressing Thrombospondin-1 (TSP-1), thereby facilitating the delivery of nutrients to cancer cells and allowing for the escape of cancer cells into the bloodstream [14,98,99]. Another player regulated by MYC in tumor microenvironment that has a distinct role in tumor angiogenesis is matrix metallopeptidase 9 (MMP-9) [24,100,101]. MMP-9 is an important accomplice of VEGF since one of its main functions is to guarantee bioavailability to VEGF [102]. As MMP-9 is also heavily involved in tumor cells’ invasion, its role will be discussed in more detail later in this review.

Moreover, it is known that some microRNAs controlled by MYC, are also involved in angiogenesis. The study of Chen and colleagues shows that miR-9 promotes angiogenesis via HIF-1α/VEGF axis and is activated by MYC in human glioma [103]. Furthermore, MYC activation in cancer cells is associated with an enhanced expression of the microRNA miR-105, which is reported to destroy vascular endothelial barriers in order to promote cancer progression and metastasis [31,104].

In a similar manner, the miR-17-92 cluster plays an important role in MYC-induced angiogenesis [105]. This cluster encodes six distinct microRNAs: miR-17, -18a, -19a, -20a, -19b, and -92a. In a mouse orthotopic xenograft model of colon cancer, Dews and colleagues demonstrated that MYC potently stimulates angiogenesis and the expression of miR-17-92 [11]. This process is partially mediated by miR-17-92 through the repression of the anti-angiogenic factors, such as TSP-1 and Connective Tissue Growth Factor (CTGF). Pro-angiogenic miR-18a and miR-19 family members, also controlled by MYC, directly target the transcripts that encode for CTGF and TSP-1, respectively, thereby reducing the expression of these anti-angiogenic proteins and allowing angiogenesis to proceed [11,106,107,108]. The pro-angiogenic effects of miR-17-92 expression are also imposed through the repression of the TGF-β signaling pathway. *TSP-1, CTGF*, and *clusterin* were among the TGF-β responsive genes whose expression was reduced in the presence of exogenous miR-17-92 [109]. The aforementioned data collectively suggests that the miR-17-92 cluster is a potent activator of angiogenesis by directly repressing anti-angiogenic factors (TSP-1 and CTGF) and targeting the TGF-β signaling pathway that leads to indirect repression of clusterin, TSP-1, and CTGF [11,108].

Mundim and colleagues show that MYC is expressed not only in breast tumor epithelial cells but also in the fibroblasts associated with primary tumor and nodal metastasis. Baudino and collaborators previously demonstrated in mouse models that MYC is a key regulator of several cytokines involved in lymphangiogenesis, such as VEGF-C and VEGF-D. This demonstrates that the increased expression of MYC can be linked with nodal metastases. Fibroblasts expressing MYC act locally at metastatic site and help to facilitate colonization by creating a lymphangiogenic microenvironment to support cancer cells’ survival [83,99]. Moreover, MYC directly regulates several genes related to glucose metabolism (such as Lactate dehydrogenase A), and CAFs were reported to have increased expression of glycolytic enzymes. Therefore, cancer cells can use these enzymes to facilitate tumor growth and vascularization [83,110,111].

In summary, all previously mentioned studies show that MYC is essential in modulating many components of the angiogenic network, both in a positive (e.g., VEGF) and inhibitory (e.g., TSP-1 and CTGF) manner. MYC is involved in a lot of different direct and indirect mechanisms of promoting angiogenesis in both cancer cells and other components of the tumor microenvironment. However, HIF-1α/VEGF axis seems to be the main axis stimulating MYC-dependent angiogenesis.

### 2.4. Immune Evasion and MYC–The Key Elements of Going under Cover

It is already established that MYC is essential in the instruction of tumor microenvironment. Tumor microenvironment is closely related to immune evasion—the mechanism through which cancer cells avoid host immune surveillance mechanisms—by inducing an immunosuppressive microenvironment. It has been recently proven that MYC favors an immunosuppressive tumor microenvironment [19]. In this section, we will explain how MYC is involved in immune response evasion and why it is crucial for tumor progression.

Reports show that KRAS and MYC collaborate in shaping the tumor microenvironment and work together to support tumor growth while avoiding immune recognition [18]. The over-expression of MYC may be one general mechanism by which tumor cells up-regulate the expression of immune checkpoint regulators, thereby evading immune surveillance [9]. MYC-driven immune evasion is the collective effort of cancer and other cells present in TME—of which TAMs are the most important ones [19,112]. Different mechanisms are explaining how immune evasion mediated by MYC could be achieved: (i) processes directly linked to expression patterns in cancer cells, (ii) crosstalk with other transcription factors, and (iii) MYC-driven recruitment of specialized and co-opted tumor-infiltrating cells [8,19,112].

Studies illustrate that high levels of MYC result in the increased expression of CD47 and PD-L1. The fact that these similar molecules are often over-expressed on the surface of human cancer cells is noteworthy. Programmed Death-Ligand 1 (or PD-L1) is a critical “don’t find me” signal directed to the adaptive immune system, whereas CD47 is an essential “don’t eat me” signal commissioned to the innate immune system. The expression of PD-L1 on cancer cells leads to evasion from the immune response, permitting cancer progression and metastasis. MYC-driven over-expression of these molecules suppress both innate and adaptive immune responses favoring tumor progression. Upon MYC inactivation, these signals are lost, and significant tumor regression is seen, confirming the relevant role of MYC in opposing anti-tumor immunity [9,113,114,115].

There is increasing evidence suggesting that MYC regulates immune response globally, as well as a study performed by Topper and colleagues showing that MYC depletion through combined epigenetic therapy reverses immune evasion enabling effective treatment of lung cancer [116]. Other works indicate that even a little increase in MYC expression is able to turn indolent KRAS-driven tumors into more aggressive, highly proliferative, and inflammatory adenomas. After MYC activation, there is a dramatic influx of CD206+ (also known as mannose receptor) TAMs in the tumor site [18]. Mannose receptors are directly up-regulated by c-MYC in tumor-associated macrophages [24]. Immunotherapy agents (like RP-182, a synthetic 10-mer peptide able to bind CD206) triggers a conformational switch of the mannose receptor CD206 expressed on M2-like macrophages and shifts the population of TAMs from M2-like macrophages toward the M1 phenotype, increasing both innate and adaptive anti-tumor immunity. These observations indicate that the MYC-dependent induction of CD206 in TAMs is important for immune evasion [117].

Furthermore, upon MYC activation, there is not only an increase in the number of macrophages infiltrating the tumor but also an immediate exclusion of T, B and NK cells within the tumor microenvironment [18]. The rapid elimination of NK cells is intriguing, as MYC activation also triggers the up-regulation of Rae-1 NKG2D ligands and the down-regulation of major histocompatibility complex (MHC) class I—both known as a potential activator of NK-like cells in lung adenoma cells [18,118].

Studies performed by Kortlever and collaborators demonstrated that these MYC-induced stromal changes are mainly driven by the up-regulation of the chemokine CCL9 (a chemokine also known as Macrophage Inflammatory Protein-1 gamma (MIP-1γ) and IL-23 in epithelial cancer cells [18,119]. Interestingly enough, it is reported that IL-23 and CCL9 are also expressed by tumor-associated macrophages [120,121,122,123], showing that immune evasion is achieved by the close collaboration between TAMs and cancer cells. To understand the individual roles of CCL9 and IL-23, Kortlever and colleagues blocked the signals separately and discovered that IL-23 mediates the rapid exit of T, B, and NK cells, but does not influence the MYC-induced macrophage influx or angiogenesis. On the contrary, the blockage of CCL9 alone inhibited MYC-induced macrophage influx, angiogenesis, and T cell loss, but did not affect NK or B cell exclusion [18].

IL-23 is a primary trigger of Th17 lymphocytes that are known to be highly immunosuppressive [18,68]. Th17 cells secrete pro-inflammatory cytokines such as IL-17A, which impair immune surveillance and promote tumor growth [124,125,126]. A few mechanisms revealing the Th17-dependent suppression of anti-tumor immunity have been proposed [127,128]. Firstly, Th17 cells are able to convert into T regulatory cells (T regs) [68,129,130]. The other reported mechanism involves TGF-β (also up-regulated by MYC). It has been demonstrated that after culturing with TGF-β/IL-6, Th17 cells co-express CD39 and CD73 ectonucleotidases on their surface. The concomitant expression of these two enzymes transforms ATP or ADP into immunosuppressive adenosine [24,128,131]. Lastly, IL-23 also serves as a potential suppressor of innate immunity most notably in NK cells; indeed, mice lacking IL-23 were more resistant to metastasis formation in models where NK cells opposed the disease’s progression [122,132].

Over the years, published reports unarguably demonstrated a crucial role for MYC in creating a favorable immunosuppressive tumor microenvironment through different mechanisms, depending on the tumor type, the tissue, and the surrounding microenvironment. Noteworthy, a significant part of these mechanisms involves the recruitment of tumor-infiltrating immune cells.

### 2.5. MYC in Invasion and Migration–First Steps in Successful Metastasis Formation

MYC up-regulates a lot of different targets involved in various mechanisms promoting tumor invasion. Here, we will briefly describe the most important ones and discuss their role in cancer cells and tumor-associated cells.

Extracellular matrix (ECM) is not just the intercellular space, but also a specific barrier for cancer cells’ migration. The degradation of the ECM is one of the most critical events in the formation of metastasis [29,133]. It has been identified that tumor cells, CAFs, and TAMs are capable of secreting a number of proteolytic enzymes, including cathepsins, and MMPs, which are essential components involved in ECM degradation and cell-ECM interactions [64,82,102,134].

MMP-9 is one key member of the MMPs family. The progression of various types of cancer, such as bladder, breast, esophageal squamous cell carcinoma, intrahepatic cholangiocarcinoma, hepatocellular carcinoma, is driven by the up-regulation of MMP-9 [135,136,137,138,139]. MMP-9 is able to cleave extracellular matrix components such as collagen or fibronectin, as well as many other non-collagenous substrates. Furthermore, MMP-9 cleavage alters chemokines’ biological functions, and its activity can result in the shedding of cell surface receptors [140,141]. A recent study shows that alternatively-activated macrophages (M2) can increase the invasion and migration of hepatocellular carcinoma by up-regulating MMP-9 [139]. Additionally, Pello and colleagues revealed that MMP-9 is induced in TAMs upon MYC activation [24,30]. It is reported that activated fibroblasts secrete increased levels of ECM-degrading proteases such as MMP2, MMP3, and MMP9, which are very important for tumor invasiveness and are potentially regulated by MYC [82,100,101,142].

Cathepsins are another family of proteins associated with different types of cancer and heavily involved in cancer aggressiveness [143]. The increase in cathepsin activity includes an enhancement in both intracellular and extracellular functions, alternative cathepsin trafficking and the recruitment of infiltrating immune cells that express and secrete cathepsins at high levels [143]. Cathepsins are mostly secreted by different immune cells, and cathepsins are reported to be up-regulated during inflammation [69,143]. Cysteine cathepsins are rapidly induced and activated in response to MYC both in cancer cells and tumor-associated macrophages [24,30,69,144]. It has been noted that in cancer cells, cathepsin L is induced by MYC; moreover, cathepsin C is known to be up-regulated by MYC in TAMs [24,144]. Secreted cathepsins are involved in extracellular matrix degradation and remodeling, and cathepsins inside the cells are part of the signaling pathways involved in cancer cell growth and inflammation [69,70]. In the past, the main focus of the studies on cathepsins in tumor invasion had been simply based on their role in ECM degradation, although the spectrum of cathepsin proteases functions is much broader. In the extracellular matrix, cathepsins can cleave different targets, starting from extracellular matrix structural components like collagen or elastin [69,143,145].

Furthermore, cathepsins are also involved in the cleavage of cell adhesion molecules (CAM), cell–cell junction disruption – all of which can influence cell adhesion and migration. What is more, proteolytic products of these cleavages can act as signaling molecules and have a role in cell growth, invasion, and angiogenesis [69,143,146]. It has been reported that secreted cathepsin B, cathepsin L and cathepsin S could cleave the cell adhesion molecule E-cadherin, promoting cancer cell invasion into the surrounding tissue [147]. Additionally, cathepsins are also involved in the processing of cytokines and chemokines, showing their involvement in inflammation and cancer processes [148,149,150].

Moreover, recent studies demonstrate that in MYC transgenic lung tumors, the gelatinase-associated protein Lipocalin 2 (LCN2) is in fact induced. This protein is a positive regulator of matrix metalloproteinases and is involved in the invasion of cancer cells [93,151]. The same study shows that, in contrast, Reck is highly suppressed upon the activation of MYC [93]. This glycoprotein is down-regulated in several tumors and serves as a negative regulator of MMPs. Hence, Reck participates in suppressing the invasiveness of cancer cells [152,153]. MYC also induces the expression of other genes, like *Adam19, Thop1, Adora2b, Hepsin*, and Amphiregulin (*AREG*) which are then involved in cancer invasion and migration. For instance, Amphiregulin up-regulates genes involved in invasion and cell motility as well as MMP-9 [93,154]. Another MYC target gene mentioned above, *CCL9*, is also known for enhancing invasion in some types of cancer [155].

Cancer-associated fibroblasts are known to play a significant role in tumor progression and metastasis also through metabolic rewiring [156,157]. Higher levels of CAFs are associated with larger tumors and shorter survival time [158]. As already mentioned, MYC activation in cancer cells results in the increased expression of miR-105, which is then packed into exosomes and transported to surrounding CAFs. In fibroblasts, miR-105 reduces the endogenous levels of MXI1, an inhibitor of MYC, and, as a consequence, increases the expression of MYC target genes in CAFs. It is important to mention that a lot of these genes augment the metabolic flexibility of CAFs so that the fibroblasts can release metabolites that could promote tumor growth and invasion [31,104]. Indeed, CAFs help cancer cells to survive and grow in an environment with high or low levels of glucose, of glutamine and pH – showing yet another example of MYC influencing tumor microenvironment and making it more congenial for cancer cells [159].

**Figure 2:**
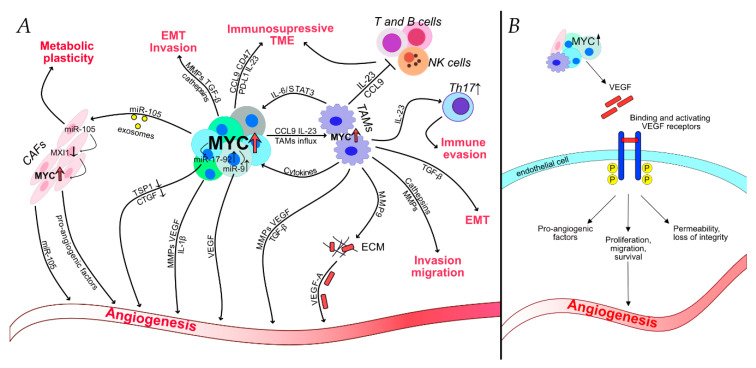
MYC and the tumor microenvironment. (**A**) MYC is an essential instructor of the tumor microenvironment [11,18,24,30,103,104]. MYC promotes cancer aggressiveness not only by influencing cancer cells’ biology but also by recruiting stromal and infiltrating inflammatory cells. The goal of this MYC-regulated crosstalk between host and cancer is to create a microenvironment that is favorable for cancer cells—facilitating growth, invasion, migration, and helping to evade the anti-tumor immune responses. (**B**) MYC controls the “angiogenic switch” [54,99]. MYC is involved in a lot of different direct and indirect mechanisms for promoting angiogenesis. MYC induces secretion of VEGF both in cancer cells and tumor-associated cells. VEGF proves to be the main factor in promoting MYC-dependent angiogenesis.

As previously mentioned, the MYC oncoprotein can provide an advantage to cancer cells by promoting proliferation, angiogenesis, and helping evade the immune response, all of which indirectly contributes to invasion and metastasis formation. However, data presented above clearly demonstrates that MYC can also directly control cellular invasion and migration, hence, metastasis, by regulating the expression of specific gene patterns and recruiting tumor-infiltrating cells (Figure 2).

## 3. MYC–The Regulator of EMT and Metastasis

Metastasis causes nearly 90% of cancer-associated deaths [160,161]. Despite the huge number of articles focusing on metastasis, the exact cellular and molecular mechanisms by which tumor cells accomplish such a complex and demanding process is not yet completely clear [162].

The formation of metastasis is a complex process and for a successful execution cancer cells must complete several complicated consecutive steps-detachment from the primary tumor, intravasation into the vascular system, survival while in transit through the circulation, initial arrest, extravasation, initial seeding, and survival and proliferation in the target tissue. Metastasis is a rather inefficient process, as large primary tumors can shed millions of cells into the blood system, but only a small number of cells are able to execute all the steps needed to form metastasis efficiently. This fact marks that in order to colonize distant organs successfully, tumor cells must acquire the necessary traits, and the environmental conditions should be favorable for the metastasis development [160,162]. One of the most important processes involved in the metastatic cascade’s crucial steps is the epithelial-to-mesenchymal transition (EMT) and is often represented as a hallmark of metastasis formation [161,162].

The direct involvement of MYC in metastasis formation is well established both in vitro and in vivo [65,163]. MYC could be one of the factors providing cancer cells with their necessary traits required for a successful metastatic progression. Previous studies demonstrate the involvement of MYC in the metastatic cascade steps. Although the role of MYC in metastasis is not questionable, MYC alone seems to be insufficient. That is why MYC cooperates with other genes and transcription factors to promote both the early (e.g., invasion and migration) and late (e.g., seeding) phases of metastatic progression. MYC is also heavily involved in the EMT process, and its direct role in metastasis will be discussed later in this review [13,14].

In this section, we will focus on the role of MYC in distant metastasis. In the previous part, we already discussed the contribution of MYC in the angiogenesis and invasion processes, which are crucial for the formation of the metastasis. Specifically, here we will discuss (a) the role of MYC in promoting metastasis through the regulation of EMT, (b) how MYC participates in the different steps of the metastatic cascade, (c) the importance of non-coding RNAs in MYC-induced metastasis as well as (d) the impact of MYC in shaping the tumor metastasis microenvironment.

### 3.1. MYC Promotes Metastasis via Regulation of EMT

The epithelial-to-mesenchymal transition (EMT) is a physiological process that generally occurs during embryonic development, tissue regeneration, organ fibrosis, and wound healing. It is a powerful action, by which epithelial cells can convert into a mesenchymal phenotype. However, it is also involved in cancer aggressiveness and, particularly, in the metastatic progression of tumors. In cancer cells, the EMT process usually does not generate terminally differentiated mesenchymal cells but creates different multiple transitional states with a mixed expression of epithelial and mesenchymal genes. EMT can be reversed through the mesenchymal-to-epithelial transition (MET). Therefore, it is believed that MET process could be important for circulating cancer cells when they reach a desirable metastatic niche to develop secondary tumors [164,165].

EMT consists of three different processes—cell–cell adhesion disruption, remodeling of the cytoskeleton, and changes in cell-matrix adhesion. What is more, EMT is also essential for the acquisition of migratory and invasive properties. The hallmark molecular marker of epithelial cells is E-cadherin, which is down-regulated during EMT, where there is a prototypical shift to N-cadherin and expression of integrins [166].

In cancers, EMT can be induced by a lot of different elements such as hypoxia, cytokines, and growth factors—all of which are secreted by the tumor microenvironment. Additionally, it has been reported that metabolic changes and immune response also play an important role in the induction of EMT.

The main regulatory elements that mediate modifications in gene expression form epithelial to mesenchymal phenotype are transcription factors (like SNAI1 and SNAI2, ZEB1 and ZEB2, Twist, and E12/E47), non-coding RNAs (miRNAs and long non-coding RNAs), epigenetic editing factors, alternative splicing processors, post-translational regulatory proteins, protein stability, and subcellular localization modifiers [167,168,169].

Experimental and clinical data suggest that there is a close relationship between the activation of EMT program and the activity of MYC transcription factor. Likewise, different reports propose that the increase in metastatic behavior of cancer cells over-expressing MYC is derived from its ability to induce cell motility through the direct or indirect modulation of the molecular markers of EMT [14,170,171].

Numerous studies show that MYC’s transcriptional signature contains genes involved in EMT. Experiments conducted by Cowling and colleagues indicate that MYC can directly down-regulate E-cadherin in breast cancer cell lines, and, as mentioned previously, the down-regulation of E-cadherin is the primary indicator of EMT [166,170]. Moreover, MYC is also known to positively regulate the expression of SNAIL—one of the master regulators of the EMT process. The work of Smith and colleagues demonstrates that MYC and TGF-β cooperate in the induction of SNAIL transcription and promote EMT in cultured epithelial cells [172]. Furthermore, the same group showed that MYC directly binds the promoter of *SNAIL* [172]. Recently, it has been reported that the SREBP1 transcription factor facilitates the binding of MYC to the *SNAIL* promoter in colorectal cancer cells, thereby accelerating *SNAIL* expression, EMT, and migration [173]. SNAIL is named the master regulator of EMT because it exerts different functions in the process. Firstly, it promotes cancer stem-like features; the EMT process supports the generation of CSCs with the phenotype of mesenchymal cells, something which happens during dissemination. It also participates in the establishment of self-renewal properties needed for initiating secondary tumors [174].

Additionally, SNAIL enhances tumor recurrence and suppresses Estrogen Receptor signaling, which leads to poor prognosis and relapses [161]. Besides, the expression of SNAIL is associated with cancer metastasis. It has also been observed that SNAIL expression levels are elevated in distant metastasis originating from ovarian cancer [175,176]. A recent study revealed that SNAIL-induced EMT accelerates metastasis through induction of immune suppression [161,177].

In a recent study, Zhao and colleagues were able to demonstrate that MYC cooperates with PIM1 to induce EMT in clear cell renal cell carcinoma. PIM1 stimulates MYC through phosphorylation leading to the up-regulation of other EMT-inducing transcription factors (ZEB1, ZEB2, SNAIL1, SNAIL2, and TWIST) [178]. This cooperation between MYC and PIM1 in inducing EMT was similarly confirmed in breast cancer cells [179].

It has been reported that EMT is significantly associated with glycans [180]. Selectins (E-, P-, and L-Selectins) are components of a family of calcium-dependent glycoproteins that are known to be involved in different steps of the metastatic cascade [181,182]. Selectins facilitate the formation of metastasis by providing cell–cell interactions between cancer and endothelial cells, cancer cells and platelets, and leukocytes. These direct interactions between different cells contribute to cancer cell adhesion, extravasation, and metastatic lesions [181]. It has been shown in colon cancer cells that during the EMT process, MYC induces the expression of sialyl Lewis x (sLex) and sialyl Lewis a (sLea) by the transcriptional up-regulation of *ST3GAL1/3/4* and *FUT3* genes [183]. sLex and sLea are E-selectin ligand glycans expressed on the surface of many types of cancer cells, including colorectal, pancreatic, gastric, breast, prostate, and lung cancer [183,184]. sLex and sLexa plays a crucial role in cancer metastasis, facilitating the extravasation of cancer cells out of the bloodstream [183,185].

Another recent study demonstrates the relationship between MYC and the oncoprotein UBE2O, a protein known to promote EMT in breast cancer cells [186]. In breast cancer cells, UBE2O up-regulates MYC through the AMPKα2/mTORC1 axis. Moreover, MYC could bind to the promoter region of UBE2O, thus transcriptionally promoting its expression and forming a positive feedback loop [186,187].

Sal-like protein 4 (SALL4) is an additional transcription factor whose increased expression promoted metastasis by the EMT process in colorectal, gastric, basal-like breast, and endometrial cancer cells [188,189,190,191]. Additionally, the recent study of Liu and colleagues demonstrates that SALL4 can induce EMT, hence, metastasis upon the deregulation of MYC. It appears that MYC is indispensable to SALL4-mediated EMT [191].

MYC can regulate both cell–cell and cell–matrix interactions through transcriptional activation of Galactin-1 (*LGALS1*) [192]. Further, it has been demonstrated that LSGAL1 promotes EMT and metastasis in lung cancers with low metastatic potential via the deregulation of α6β4 Integrin and Notch1/Jagged signaling pathways [193].

Interestingly, studies have shown that MYC is not only directly involved in EMT promotion but that it also plays a crucial role in the epigenetic regulation of the expression of EMT promoting transcription factors (EMT-TFs). MYC can specifically recognize the E-box motifs present in the regulatory regions of *SNAIL, ZEB1*, and *ZEB2* pro-EMT genes and is responsible for the induction of their expression [194,195]. This up-regulation can be mediated by the MYC-dependent recruitment of multiple epigenetic modulators. In addition, MYC has a crucial role in switching between active and repressive components in the epigenetic regulation of pro-EMT transcription factors [195].

### 3.2. MYC’s Involvement in the Metastatic Cascade

The formation of metastasis is a complex process involving the local invasion of cancer cells, intravasation, circulation of tumor cells, and extravasation into distant organs. MYC seems to be involved in most of these steps, both directly and by the cooperation with other oncogenes [14,162].

Previous studies show that MYC cooperates with c-RAF to induce lung cancer metastasis [65,196]. Moreover, RAS activates a signaling cascade that leads to MYC protein stabilization in cancer cells. MYC, in turn, is required for the maintenance, progression, and metastasis of RAS-driven tumors such as lung cancer [14,197]. Furthermore, MYC and TWIST1 cause metastasis by switching a transcriptional program in cancer cells that induces cytokines. Cytokines enable the crosstalk between host and tumor cells and stimulate the recruitment and polarization of macrophages. MYC and TWIST 1 coordinate to regulate a cytokinome, including CCL2 and IL-13, that are both necessary and sufficient in order to promote the formation of metastasis [25].

Breast cancer cells-derived brain metastases exhibit an increased MYC activity. The brain is a specialized microenvironment, and MYC can support the formation of metastases in that district not only by promoting invasive growth but also by cross-talking with TME. It has been reported that in brain metastases derived from breast cancer the up-regulation of MYC participates in the recruitment of macrophages and plays an important role in the formation of gap junctions between metastatic cells and astrocytes, which seems crucial for the metastatic growth within the brain [198]. MYC takes part in the generation of gap junctions through the up-regulation of Connexin 43 (Cx43), a crucial protein in favoring close interactions between cancer cells and endothelial cells, hence, facilitating extravasation and local survival of metastatic cells [198,199]. The correlation between the up-regulation of Cx43 and metastasis is reported not only in breast cancer, but also in prostate cancer, where the increased expression of Cx43 is involved in bone-specific metastasis, the most common target site for advanced prostate cancers [200].

Osteopontin (OPN) is a cytokine and integrin-binding ligand that is positively regulated by MYC [201] and that is known to have pro-metastatic functions in cancer [202]. Allan and colleagues described OPN as a key molecular player involved in lymphatic metastasis originating from breast cancer [203]. Additionally, OPN is produced not only by cancer cells but also by stroma and infiltrating inflammatory cells, and is reported to have multiple effects in the specific steps of metastatic cascade [204,205]. Various studies describe that in breast cancer, OPN facilitates cancer cell detachment and intravasation by inducing the expression of EMT promoting transcription factors including TWIST, SNAIL, SLUG, and MMPs. Furthermore, OPN is important for the circulating tumor cells as it helps to evade immune cells by suppressing immune responses [205,206,207]. Recent data shows that MYC is also over-expressed in circulating tumor cells (CTSs), suggesting that its target genes could be essential for the survival of cancer cells in the bloodstream [208].

Alternatively, the study by Chan and colleagues demonstrates that MYC can cooperate with SKP2 to recruit MIZ1 and p300 forming a complex that activates Rho A [209]. RhoA is over-expressed in several cancers and is implicated in metastasis. RhoA participates in the formation of invadopodia which are known to enhance tumor metastasis by disrupting the basement membrane [210,211].

Similarly, the over-expression of MYC in metastatic tumor cells leads to the up-regulation of Id2 and, hence, to the repression of SEMA3F. SEMA3F is a potent inhibitor of metastasis, and, therefore, its repression enhances tumor dissemination [212,213,214].

### 3.3. Non-Coding RNAs Plays an Integral Role in MYC-Mediated Metastatic Progression

As previously mentioned in above sections, MYC controls a lot of different pro-tumorigenic functions not only by directly targeting the genes of interest but also by controlling a wide range of non-coding RNAs–microRNAs and long non-coding RNAs [105,215]. It seems that in addition to the transcriptional regulation of protein-coding genes, miRNAs are the integral nodes in the transcriptional network controlled by MYC. The deregulation of MYC has been reported in metastasis, and MYC-regulated ncRNAs play an essential role [105,108,216].

Specifically, MYC-stimulated miR-9 was shown to directly repress E-cadherin in a breast cancer model [217,218]. Moreover, it has been shown that the increased expression of miR-9 has pro-metastatic properties. For example, the over-expression of miR-9 in previously non-metastatic breast cancer cells led to the formation of micrometastases in mice [217]. Furthermore, MYC and its family member MYCN directly induce miR-9 expression in breast cancer and neuroblastoma models. From what is currently known, the repression of E-cadherin through the MYC/miR-9 axis has a critical role in the development of metastatic cancers, as it influences angiogenesis, EMT, and invasion [219].

As previously mentioned, MYC can directly regulate the epithelial-to-mesenchymal transition by promoting TGF-β-mediated activation of SNAIL; however, this regulation can also be indirect, and MYC can additionally act through a non-coding RNA network involving a LIN28B/let-7/HMGA2 cascade [172,220,221,222,223,224]. Indeed, MYC can activate the transcription of LIN28B, which is responsible for the repression of let-7 and, consequently, HMHA2 that, in turn, results in the up-regulation of SNAIL, increased invasiveness, and enhanced metastatic potential. Additionally, LIN28B is reported to promote metastasis in colon and ovarian cancers, and some studies claim that MYCN could be directly responsible for the deregulation of LIN28B [108,225,226].

miR-200 is another microRNA heavily involved in the regulation EMT process that is affected by MYC levels. The miR-200 family of microRNAs directly targets the 3’UTR of both ZEB1 and ZEB2, transcription factors that inhibit E-cadherin expression [227,228,229,230]. MYC protein levels have been inversely correlated with miR-200c expression in nasopharyngeal cancer primary tumors. Specifically, MYC has been shown to directly repress miR-200 in a transcription-dependent fashion [231]. Furthermore, miR-200c reduces the expression of MYC by binding to its 3’-untranslated region, suggesting the existence of a negative feedback loop between MYC and miR-200c. The over-expression of MYC interferes with this feedback loop, and the repression of miR-200 results in the induction of EMT, dependent on the up-regulation of ZEB1 and ZEB2 [108,231].

Wang and colleagues investigated the relationship between MYC and miR-148a-3p, whose over-expression inhibits cell motility and EMT. They reported that upon MYC over-expression, miR-148a-3p levels decreased, leading to increased EMT, migration, and invasion [232,233].

Equally important, it has been proposed that MYC can activate miR-105 [31]. Increased levels of circulating miR-105 and miR-122 can be detected at a pre-metastatic stage and correlate with the occurrence of metastasis in breast cancer patients [31,104,234]. A recent study trying to understand whether miR-105 can be considered an oncogene or a tumor suppressor shows contradictory results [235]. However, in colorectal cancer, the up-regulation of miR-105 promotes epithelial-mesenchymal transition and metastasis both in vitro and in vivo. At the molecular level, miR-105 directly targets the 3’UTR of RAP2C (also as a result of the activation of the NF-κB signaling pathway), a small GTPase whose over-expression reduces the migratory and invasive ability of colorectal cancer cells [236]. Namely, studies on breast cancer demonstrate that metastatic breast cancer cells secrete a higher level of miR-105 than non-cancerous mammary epithelial cells. The same work reports that miR-105 is an important regulator of migration through the targeting of the tight junction protein ZO-1, leading to the disruption of tight-junction barriers and the promotion of metastasis in vitro and in vivo [104,235].

MYC is also responsible for the regulation of some lncRNAs involved in the EMT process and metastasis formation [216]. A novel lncRNA, HOXC13-AS, was described as an oncogene in gliomas. The down-regulation of HOXC13-AS repressed the migration, invasion, and EMT process of glioma cells. Moreover, Liu and colleagues demonstrate that MYC binds the HOXC13-AS promoter region and activates this long non-coding RNA, which therefore leads to increased migration, invasion, and EMT [237].

Recent experimental results on breast cancer cell lines indicate that LINC01433 is activated by MYC and promotes cell proliferation, migration, and the epithelial-mesenchymal transition by sponging miR-2116-3p as a mean to up-regulate MYC [238]. Further, a recent study reveals that the knockdown of the long non-coding RNA HOTTIP inhibits cell proliferation, migration, and significantly suppresses the expression of glycogen synthase kinase 3β, β-Catenin and MYC in colorectal cancer [239]. A similar study demonstrated that lncRNA HOTTIP was over-expressed in osteosarcoma cells and may facilitate migration, invasion, and EMT in vitro by forming a positive feedback loop with MYC [240]. Nevertheless, the over-expression of MYC, in turn, increased HOTTIP expression positively feeding the circuit. MYC over-expression may promote osteosarcoma cell migration and invasion in vitro, which may be related to the pro-metastatic role of HOTTIP in osteosarcoma cells [240]. Furthermore, studies in colorectal cancer cells show that HOTTIP regulates metastasis partly through the down-regulation of the tumor suppressor DKK1 [241].

Additionally, it is reported that in non-small lung cancer cells, MYC up-regulates the long non-coding RNA BCYRN1. BCYRN1 is responsible for the up-regulation of MMP-9 and MMP-13. MMP9 is most likely involved in the initial degradation of the basement membrane surrounding the primary tumor, and MMP13 has a broader substrate specificity than other collagenases and can cleave type IV, X, and XIV collagens, tenascin, aggrecan core protein, and fibronectin. This observation demonstrates that MYC-activated lncRNA BCYRN1 has the ability to regulate the metastatic spread of cancer cells through the up-regulation of MMP9 and MMP13 [242].

The reprogramming of non-coding RNA expression mediated by MYC reveals an intriguing and complex way this oncoprotein exploits to influence different pathological processes. The network of MYC-controlled ncRNAs regulates key elements of the oncogenic program, promoting tumor progression and metastasis; we have summarized them in Table 2.

### 3.4. MYC Recruits TAMs to Promote EMT and Metastasis

The tumor microenvironment is essential for malignant tumor progression and metastasis formation [243]. TAMs are reported to be pivotal players in tumor aggressiveness. Biologically, macrophages mediate adhesion, facilitate the EMT process in cancer cells and promote distant metastasis spread via secreting various soluble factors, such as IL-1β, IL-8, TNF-α, TGF-β, MMP-9. More importantly, the expression of some of these molecules is induced by MYC [24,29,64,244,245,246]. It has been reported that in TAMs, MYC is responsible for the up-regulation of MMP-9 [24].

To this end, a recent study demonstrates that MMP-9 secreted by tumor-associated macrophages promotes metastasis (via EMT) through the activation of the PI3K/AKT signaling pathway [64]. It has been shown that TAMs are also crucial in establishing a pre-metastatic microenvironment in distant organs [247,248]. Tumor-associated macrophages forming the pre-metastatic niche are recruited even before the arrival of tumor cells via the production of specific cytokines, and the primary tumor controls this whole process. These TAMs within the pre-metastatic niche facilitate tumor cell extravasation, seeding, survival, and subsequent colonization on the secondary site [249]. Currently, there is no data that directly shows MYC’s functions in the pre-metastatic niche formation, however, knowing the potential of MYC in recruiting macrophages and that it is a master regulator of the tumor microenvironment, we can suspect that it is only a matter of time until a role for MYC in pre-metastatic microenvironment will be recognized.

**Figure 3:**
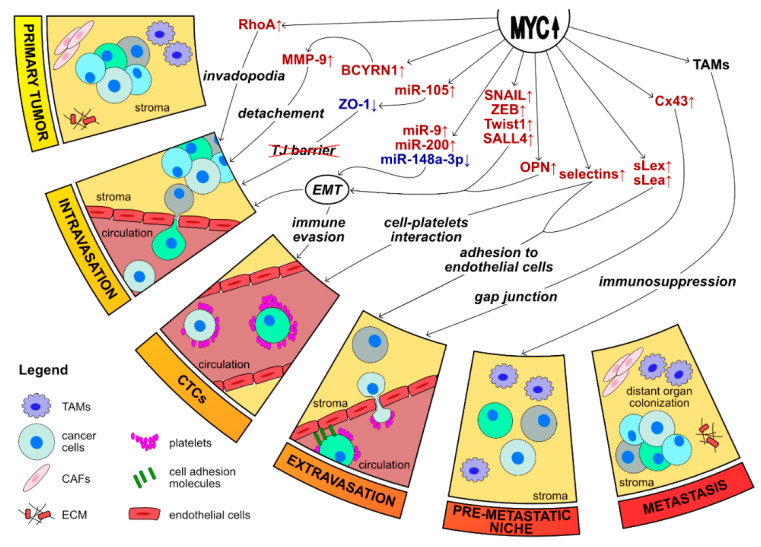
MYC is an important player in the formation of metastasis. Metastasis is a complex process involving the detachment from the primary tumor, intravasation into the bloodstream, survival within circulation, arrest, extravasation, and colonization at distant sites. MYC regulates metastasis formation using several different approaches—directly, co-operating with other oncoproteins, epigenetically, using a network of ncRNAs or recruiting components of the microenvironment. EMT seems to be at the center of MYC-induced metastasis [13,24,25,31,112,172,178,179,183,191,242].

All in all, metastasis formation is a very complicated process, and the mentioned data suggests that MYC has a role in every step of this process, we have summarized in Figure 3. EMT is the central program in MYC-dependent metastasis formation. MYC can regulate this process from a lot of different angles – directly, co-operating with other oncoproteins or also epigenetically. Moreover, an increasing number of studies demonstrate that MYC can regulate EMT and metastasis via modulating non-coding RNAs. These observations reveal yet another way in which MYC is promoting tumor progression. Lastly, MYC seems to be able to successfully recruit active components of the tumor microenvironment for its needs, and EMT and metastasis formation are not an exception.

## 4. Conclusions

To summarize, MYC is undoubtedly an important player in cancer aggressiveness and progression. MYC promotes angiogenesis, immune evasion, invasion, and migration, hence, metastasis directly by regulating the expression of specific gene patterns or by cooperating with other transcription factors as well as by controlling a network of non-coding RNAs. Moreover, MYC is able to successfully support tumor progression by orchestrating active crosstalk between cancer cells and the host. Because of the ability to recruit stromal and infiltrating immune cells to promote invasive growth, MYC could also be called “the master regulator of tumor microenvironment”. Although MYC is a highly desirable target for anti-cancer drug development, there has been a lot of challenges that have held back the development of drugs directly targeting MYC [250,251]. However, there are already reported studies successful inhibiting MYC in tumor-associated macrophages, demonstrating that a better understanding of the role of MYC in the tumor microenvironment and metastasis could represent an effective way to successfully target MYC in cancer [30,252].

## Figures and Tables

**Table 1:** Cytokines, chemokines, enzymes and signaling molecules up-regulated by MYC in tumor-associated macrophages (TAMs) and their role in cancer aggressiveness and progression.

	Processes	Mechanisms	References
MMP-9	Invasion, migration Angiogenesis EMT	Remodeling and degradation of ECM Release VEGF from ECM PI3K/AKT signaling pathway	[24,64]
VEGF	Angiogenesis	Blood vessel permeability, loss of integrity	[17,24,65]
TGF- β	EMT	via SMAD/SNAIL	[24,64]
ALOX15	Alternative macrophage activation	M2/TAMs marker	[24,66]
	Immunosuppression	via IL-10/CCL-2	
HIF-1α	Angiogenesis	HIF-1α/VEGF axis	[24,67]
	Immunosuppression	Macrophage recruitment	
CCL9	Immune evasion	Macrophage influx, T cell loss	[18]
	Angiogenesis	via VEFG	
IL-23	Immune evasion	Expulsion of T, B and NK cells, Th17 influx	[18,68]
CTCS	Invasion	Remodeling and degradation of ECM	[24,69,70]
STAT6	Alternative macrophage activation	Downregulation of TRIM24	[24,71]
CD209	Alternative macrophage activation	M2/TAMs marker	[24,72]
MRC1	Alternative macrophage activation	M2/TAMs marker	[24,73]
PPARγ	Alternative macrophage activation	M2/TAMs marker	[24]
SCARB1	Alternative macrophage activation	M2/TAMs marker	[24]

**Table 2:** The role of non-coding RNAs regulated by MYC involved in cancer aggressiveness and metastasis. A brief summary of non-coding RNAs controlled by MYC, the processes they influence, and the respective targets/signaling pathways mediating these processes.

	Processes	Target/Signaling Pathways	References
*microRNAs activated by Myc*			
miR-17-92 cluster:			
miR-18a miR-19	Angiogenesis	CTGF ↓ TSP-1 ↓	[11,106,107,109]
miR-105	Metabolic rewiring Angiogenesis Metastasis, EMT	MIX1 ↓ ZO-1 ↓ ZO-1 ↓ RAP2C ↑	[104,136,137,235,236]
miR-9	Angiogenesis Metastasis, EMT	HIF-1α/VEGF axis E-Cadherin ↓	[103,218]
*microRNAs suppressed by Myc*			
miR-148a-3p	EMT	ERBB3/AKT2/MYC axis	[233]
miR-200	EMT	Zeb-1 ↑ Zeb-2 ↑	[108,231]
*long non-coding RNAs activated by Myc*			
HOXC13-AS	Migration, invasion, EMT	MYC ↑	[237]
LIN28B	EMT	let-7 ↓ HMHA2 ↓ Snail ↑	[172,221,222,223,224]
HOTTIP	Migration, invasion, EMT Metastasis	MYC ↑ β-catenin ↑ Vimetin ↑ DKK1 ↓	[239,240,241]
LINC01433	Migration, EMT	Myc ↑ (via sponging miR-2116-3p)	[238]
BCYRN1	Metastasis	MMP-9 ↑ MMP-13 ↑	[242]

## Abbreviations

EMT	Epithelial-to-Mesenchymal Transition
TME	Tumor MicroEnvironment
TAMs	Tumor-Associated Macrophages
CAFs	Cancer-Associated Fibroblasts
ECM	ExtraCellular Matrix
NK	Natural Killer
miR	microRNA
lncRNA	long non-coding RNA
